# Widening socioeconomic inequalities in cancer incidence and related potential to reduce cancer between 2008 and 2019 in Germany

**DOI:** 10.1038/s41598-025-17859-5

**Published:** 2025-09-01

**Authors:** Fabian Tetzlaff, Benjamin Barnes, Lina Jansen, Frederik Peters, Annemarie Schultz, Alexander Katalinic, Klaus Kraywinkel, Niels Michalski, Enno Nowossadeck, Jens Hoebel

**Affiliations:** 1 Division of Social Determinants of Health, Department of Epidemiology and Health Monitoring, Robert Koch Institute, Nordufer 20, 13353 Berlin, Germany; 2 German Centre for Cancer Registry Data, Department of Epidemiology and Health Monitoring, Robert Koch Institute, Berlin, Germany; 3 Epidemiological Cancer Registry of Baden-Württemberg, German Cancer Research Center (DKFZ), Heidelberg, Germany; 4 Cancer Registry Hamburg, Hamburg, Germany; 5 Institute for Social Medicine and Epidemiology, University of Lübeck, Lübeck, Germany

**Keywords:** Trends cancer incidence, Area-level socioeconomic inequalities, GISD, Germany, Deprivation, Social determinants, Cancer epidemiology, Cancer epidemiology

## Abstract

Background Cancer is one of the main causes of a high burden of disease and one of the strongest contributors to earlier mortality among lower socioeconomic groups in Germany. Therefore, studying socio-economic inequalities in cancer incidence is of high relevance from a public-health and health-equity lens. The aim of this study was to examine in more depth time trends in socioeconomic inequalities in cancer incidence and the related potential for reducing the incidence of specific cancers across Germany. Methods We used epidemiologic data from the Centre for Cancer Registry Data at the Robert Koch Institute and official population statistics for Germany from 2008 to 2019. To analyse trends in socioeconomic inequalities in cancer incidence, we used an ecological study design and linked the cancer registry and population data with the German Index of Socioeconomic Deprivation at district level. We calculated standardised cancer incidence rates for the most common cancers by area-level socioeconomic deprivation and estimated the Slope and Relative Index of Inequality (SII, RII) to determine the extent of area-level socioeconomic inequalities in the risk of cancer. In a what-if analysis, counterfactual scenarios were used to calculate how much lower cancer incidence could be if socioeconomic inequalities in incidence were reduced or eliminated. Results Due to less favourable trends of cancer incidence in more deprived areas, socioeconomic inequalities in cancer incidence has widened to the detriment of residents in highly deprived areas. This was observed for all cancers combined and for several common cancers such as stomach, colorectal and lung cancer among both women and men. In 2017–19, total cancer incidence was 18% (women: RII 1,18) and 49% (men: RII 1,49) higher in the most than in the least deprived area. Reverse inequalities were observed for skin melanoma in both sexes and female breast cancer, the lowest incidence being among residents of highly deprived districts. For 2017–19, the what-if analysis showed that the annual number of newly diagnosed cancers cases would be 9,100–76,000 cases fewer if the socioeconomic gap in cancer incidence between districts could be narrowed or eliminated. Conclusions In Germany, socioeconomic inequalities in cancer incidence have widened in recent decades. Tackling cancer risks in deprived areas could reduce those inequalities and the burden of cancer overall. Our study emphasises the growing importance of structural approaches in cancer prevention for reducing health inequalities in Germany.

## Introduction

Cancer is one of the main causes of premature mortality and leads to a high burden of disease in Germany^1–3^. Cancer also represents one of the strongest contributors to the socioeconomic gap in life expectancy in Germany, i.e. more premature deaths among people from lower socioeconomic circumstances^2^. Reducing inequalities in morbidity and mortality is a major objective of public health. So far, however, the evidence on how social inequalities in cancer incidence in Germany have developed over time is still scarce, and there is little research on how many cancer cases could be reduced by eliminating socioeconomic inequalities in cancer incidence. In this study, we aim to examine in more depth time trends in socioeconomic inequalities in cancer incidence and the related potential for reducing the incidence of specific cancers for Germany.

In general, the relationship between socioeconomic position and severe diseases such as cancer can be viewed from two causal directions: cancer may be the cumulative result of exposure to risk factors associated with low socioeconomic position^4^, and functional limitations due to the disease can lead to downward social mobility after a cancer diagnosis^5^, both of which can lead to the health inequality observable in a population. For many European countries, pronounced socioeconomic inequalities in cancer-related morbidity (e.g.^6–8^), mortality (e.g.^6,9^), and survival (e.g.^10^) have been demonstrated for the most common cancers. This also applies to Germany^1,2,11–15^. However, findings on the development of socioeconomic inequalities in cancer incidence over time at national level are scarce due to a lack of available data on the socioeconomic position (SEP) of individuals with cancer. This is mainly due to the fact that the cancer registries in Germany do not gather individual-level information on SEP. A common strategy is therefore either to use data from the statutory health insurers^12^ or to apply an ecological study design using area-level socioeconomic information^13,14^. Due to cancer’s high morbidity burden and case-fatality rate, cancer prevention remains a key goal in health policies. This study therefore analyses for the first time how many cancer cases in Germany could potentially be reduced if socioeconomic inequalities in the incidence at area level could be narrowed or eliminated.

One of the first studies analysing socioeconomic inequalities in cancer incidence on a national scale for Germany reported substantial differences by area-level socioeconomic deprivation for various cancers^13^. The largest socioeconomic inequalities were reported for lung cancer and colorectal cancer, with higher incidence rates observed in highly deprived areas. These findings were supported by trend analyses based on cancer registry data^14^ and claims data of statutory health insurers^12^. In these studies, widening socioeconomic inequalities for colorectal and lung cancer were reported^12,14^. However, both of these studies were limited to specific population groups (the western federal states of Germany or an insurance population concentrated in the federal state of Lower Saxony). Our study goes further and includes also the eastern federal states, where a large proportion of Germany’s most deprived areas are located^16^. In contrast to previous studies^12–14,17^, we are therefore able to provide a more detailed picture of the development of socioeconomic inequalities in cancer incidence in Germany. Our study aims to analyse time trends in area-level socioeconomic inequalities in the incidence of the most common cancers for thirteen of the sixteen German federal states.

Our study addresses the following questions:

1. How large is the gap in area-level socioeconomic deprivation in the incidence of the most common cancers in Germany?
2. How has the incidence of common cancers developed in more and less deprived areas in recent decades?
3. Did area-level socioeconomic inequalities in the most common cancers change in their extent between 2008 and 2019? Did inequalities widen or narrow?
4. To what extent could the number of new cases of the most common cancers in Germany be reduced if area-level socioeconomic inequalities in cancer incidence could be reduced or eliminated?

## Methods

### Data

Our study was based on the epidemiological data from the Centre for Cancer Registry Data at the Robert Koch Institute (RKI)^18,19^ and the official population numbers for the German districts from 2008 to 2019^20^. In addition to sociodemographic characteristics (sex and age at the time of diagnosis) and diagnosis codes according to the International Statistical Classification of Diseases, 10th revision, clinical modification (ICD-10-CM), the data also included information on the place of residence of the registered persons at the time of diagnosis. Using this data, it is common practice to only include the data of cancer registries that have achieved sufficient completeness of the recorded cancer cases (e.g.^13,14^). In order to include as many registries and calendar years as possible, we divided the data into four three-year periods (2008/10, 2011/13, 2014/16, 2017/19) with an average estimated completeness of registries of 90% for each period. This approach allowed us to include data from 13 of the 16 German federal states (excluding Baden-Württemberg due to missing data at the beginning of the observation, and Saxony-Anhalt and Thuringia due to low estimates of completeness). Cases identified by death certificates only were also included, using the date of death as an approximation of the date of incidence^13,14^. Death certificates are a routine source of information for all cancer registries in Germany. For death certificates that mention cancer, a linkage with previously recorded cancer registry data was conducted using personally identifying information such as names, addresses and birth dates. This linkage took place in the federal state in which the deceased person lived before we accessed the data, and information from death certificates regarding previously registered cases is integrated into the existing registry data. In this way, the likelihood of double registration of DCO cases is reduced. Furthermore, we focused on middle and older adulthood, as cancers in childhood, adolescence and young adulthood often have a different etiology and are attributable to other causes or risk factors than cancers after the age of 40. We focused on the most common cancers and those cancer entities for which a social gradient has been reported in previous studies for Germany^12–14,17^. These are tumours of the mouth (C00–06), stomach (C16), colorectum (C18-20), pancreas (C25), lung (C33-34), skin melanoma (C43), female breast (C50), cervix uteri (C53) and prostate (C53). Furthermore, we analysed only diagnoses of primary malignant neoplasms. Secondary tumours (C77-79) and diagnoses of in situ neoplasms, benign neoplasms, neoplasms of uncertain or unknown behaviour (D00-D48), and diagnoses of non-melanoma skin cancer (C44) were excluded from our analyses.

### Area-level socioeconomic deprivation

The data from the German cancer registries do not contain any individual-level information on SEP. We therefore used an ecological study design. To approximate SEP at a small-area level, we used the information on the place of residence of the registered persons at the time of diagnosis. On this basis, we linked the cancer registry data with the German Index of Socioeconomic Deprivation (GISD)^16,21^ at district level (German ‘Kreise’ equivalent to level 3 of the Nomenclature des Unités territoriales statistiques NUTS); *N* = 320 districts; Table 1), and used the averages of the GISD of the three-year time periods for our trend analysis. The GISD is a composite index to measure the relative socioeconomic deprivation of all regions in Germany and is based on nine spatially aggregated indicators reflecting the three core dimensions of SEP (education, employment, income) and has proven to be suitable for investigating socioeconomic inequalities in cancer-related outcomes^2,13,22–24^. Further details on the methodology of the GISD can be found in Michalski et al.^16^.

**Table 1:** Description of the study population in total and by GISD quintile, 2008–2019.

	Total	Women	Men
Mean population size per year	41,410,972	21,733,851	19,677,121
–Deprivation quintile 1 (least deprived)	8,928,201	4,671,855	4,256,346
–Deprivation quintile 2	7,974,577	4,185,973	3,788,605
–Deprivation quintile 3	7,937,411	4,153,036	3,784,376
–Deprivation quintile 4	7,362,145	3,880,021	3,482,124
–Deprivation quintile 5 (highly deprived)	9,208,638	4,842,967	4,365,671
Average number of incident cancer cases^a^ per year	432,344	203,638	228,706
–Deprivation quintile 1 (least deprived)	87,242	42,216	45,027
–Deprivation quintile 2	82,783	39,191	43,592
–Deprivation quintile 3	83,534	39,070	44,464
–Deprivation quintile 4	79,992	37,101	42,891
–Deprivation quintile 5 (highly deprived)	98,793	46,060	52,733
Number of first-level units in the dataset^b^	76,800	38,400	38,400
Number of districts (second-level units)	320	320	320

^a^For all cancers combined (C00–C97 excluding C44 and C77–C79); ^b^Product of the number of age groups (*n* = 10), districts (*n* = 320), observation years (*n* = 12), and sexes (*n* = 2)

Data: annual epidemiological data from the Centre for Cancer Registry Data at the RKI, population statistics of the Federal Statistical Offices, GISD, own calculations

**Fig. 1:**
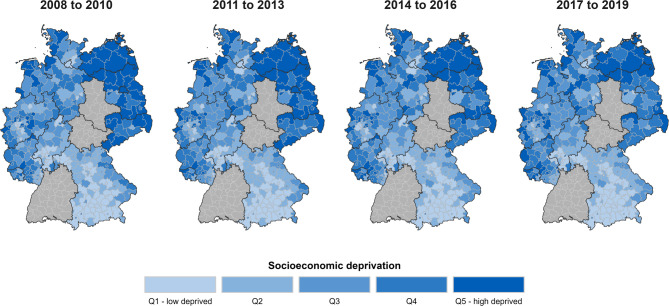
Maps of Germany’s districts by area-level socioeconomic deprivation (quintiles) for regions covered by the included cancer registries between 2008–2019. Data: GISD; Projection: Gauss-Krueger 3 (EPSG:31467); Scale: 1:2.500.000; GeoData: © GeoBasis-DE/BKG 2023; Software: R 4.4.1 (the code to generate the figure is available on request).

To assess inequalities in cancer incidence by regional socioeconomic deprivation, we divided the included districts according to their GISD score into quintiles (*n* = 64 districts per quintile) and categorised the quintiles as ‘low deprivation’ (quintile 1), ‘middle deprivation‘ (quintiles 2–4) and ’high deprivation‘ (quintile 5). The assignment of the included districts to the quintiles of socioeconomic deprivation can be found in Fig. 1. The highest levels of socioeconomic deprivation occur primarily in the north-east of Germany, in the urban regions of North Rhine-Westphalia, and in the federal states of Rhineland-Palatinate and Saarland. The lowest levels of deprivation are found in the south of Germany.

### Statistical analysis

This study is based on two analytical steps:

First, we calculated directly age-standardised incidence rates per 100,000 persons and 95% confidence intervals^24^ separately by sex, area deprivation and time period. To ensure the international comparability of our results, we used the 2013 European Standard Population^25^ for persons aged 40 or older (five-year age groups with a collapsed upper age group of 85+) for standardisation. To quantify the magnitude of area-level socioeconomic inequalities in cancer incidence in absolute and relative terms, we calculated the Slope Index of Inequality (SII) and the Relative Index of Inequality (RII) via^27,28^ applying Poisson regression models. In these models, the number of incident cancer cases registered within each age group of a district was regressed on the district’s level of socioeconomic deprivation (GISD score ranging from 0 [least deprived] to 1 [most deprived]), with age groups as co-variables. Since the districts were heterogeneous in relation to their population composition (age structure) and the data were clustered and hierarchically structured (age groups nested within districts), the models were fitted as multi-level models with age groups as first-level and districts as second-level units. We used the age-specific standard population as weights and the logarithm of the age-specific population numbers of the districts as offset term in the estimation. SII and RII analyses were stratified by cancer site, sex, and time period.

Second, a what-if analysis was conducted with three different counterfactual scenarios to quantify how much lower site-specific cancer incidence could be if area-level socioeconomic inequalities in cancer incidence were reduced or eliminated. These scenarios took the perspective of the ‘levelling up’ approach^29,30^, i.e. equalising the health and life chances of groups with lower SEP to the levels of the groups with higher SEP. This approach assumes increased health equity through improvements of health and life chances in disadvantaged socioeconomic groups. Accordingly, we restricted the what-if analysis to cancers showing a social gradient with higher incidence rates in more deprived areas. Cancers with a reverse social gradient were excluded from the what-if analysis (breast cancer, cervix uteri, prostate cancer and skin melanoma) because including them would have violated the aforementioned assumption.

The scenarios were based on the following assumptions:

Scenario 1: Residents of all districts in Germany have the same cancer incidence as residents of the least deprived districts (levelling the age-specific incidence in deprivation quintiles 2 to 5 to the age-specific incidence rates in deprivation quintile 1).

Scenario 2: The cancer incidence in more deprived districts improves to the incidence level in the next less deprived districts (levelling the age-specific incidence in deprivation quintiles 2 to 5 to the age-specific incidence of the next lower deprivation quintile).

Scenario 3: The cancer incidence in districts with above-average deprivation improves to the incidence level of districts with middle deprivation (levelling the age-specific cancer incidence in deprivation quintiles 4 and 5 to the age-specific cancer incidence of deprivation quintile 3).

The expected incident cancer cases ($\:\widehat{IC}$) for each scenario were calculated by multiplying the population in age group *i* and deprivation quintile *j* in the included districts ($\:{N}_{ij}$) by the age-specific incidence in the corresponding GISD quintile for the respective scenario j* ($\:{ASIR}_{ij*}$) for each cancer entity:

$$\:\widehat{IC}={\sum\:}_{j=1}^{5}{\sum\:}_{i=40}^{85+}{N}_{ij}*{ASIR}_{ij*}$$

The potentially reduceable cancer cases ($\:PRIC$) resulted from the difference between the sum of the expected cancer cases ($\:\widehat{IC}$) and the observed cancer cases ($\:IC$) for each cancer entity:

$$\:PRIC=\widehat{IC}-{\sum\:}_{40}^{85+}IC$$

In order to determine the extent to which the site-specific incidence could be reduced in relative terms, the proportion of incident cases counterfactually reduced in the scenario in relation to all observed site-specific new cases was also calculated. The analyses were performed using STATA 17 and R 4.1.3. We adhered to the Strengthening the Reporting of Observational Studies in Epidemiology (STROBE) statement to report study results^31^.

### Sensitivity analysis

To check for robustness, we replicated our analysis with different inclusion criteria. First, we excluded municipalities in Germany that represented large cities, a district or an entire federal state (Berlin, Hamburg and Bremen). This was done because the spatial resolution for these districts is very crude, as the data do not allow any further breakdown within these cities, e.g. by city districts. Moreover, these districts have a very large and socioeconomically heterogeneous population^32^. Therefore, it can be assumed that the inequalities analysed in the main analyses are smaller than they would be if these districts could be included at finer resolutions.

Second, since the risk of developing cancer accumulates over the life course before incidence occurs in most entities, it could be argued that the long-term effect of socioeconomic deprivation on cancer incidence may be more important than the current regional socioeconomic deprivation. To consider this aspect, we used the GISD score of the respective districts measured 10 years prior to the date of incidence to calculate the inequalities in site-specific cancer incidence under the assumption that persons diagnosed with cancer did not live in a district of a different deprivation quintile in this time period.

## Results

Despite the exclusion of three registries, we were able to include around 81% of the population in Germany aged 40 and over, and to rely on data from around 41.4 million people at risk of cancer and on average 432,000 incident cancer cases per year (Table 1). The age structure of the population at risk and of the incident population differ not markedly between the deprivation quintiles (Table S2).

Table 2 depicts the area-level socioeconomic inequalities in cancer incidence among residents of the quintiles of districts in Germany for the period 2017–2019. For both sexes, age-standardised incidence rates in the most deprived quintile of districts were higher than in the least deprived quintile of districts for all cancers combined, mouth cancer, colorectal cancer, lung cancer, stomach cancer, and other cancer. This also applies to pancreatic cancer in men. Furthermore, we found consistently higher site-specific incidence rates and more pronounced area-level socioeconomic inequalities in cancer incidence among men than women. The latter was true for both absolute and relative inequalities. Due to the stronger aggregation, the inequalities between quintiles of area-level deprivation are smaller compared to SII and RII analysis, but they largely point in the same direction. Considering the whole range of area-level socioeconomic deprivation, the incidence of colorectal cancer in men was approximately 60% (RII = 1.57) higher among residents of most deprived districts compared to less deprived districts. The strongest socioeconomic inequalities were found in cancer of the mouth (women: RII = 1.84; men: RII = 2.78) and lung cancer (women: RII = 2.20; men: RII = 2.75). A reversed gradient was found for breast cancer in women and malignant melanoma of the skin in both sexes. For cervix uteri and prostate cancer, no inequality was found according to area-level socioeconomic deprivation. Overall, when considering the category of all cancers combined, cancer incidence was 18% (women: RII 1,18) and 49% (men: RII 1,49) higher in the most deprived areas than in the least deprived ones.

**Table 2:** Age-standardised cancer incidence at age 40 + by sex, quintiles of area-level socioeconomic deprivation, and cancer entity in the period 2017–2019.

	Incidence rates per 100,000 residents and year					Summary measures of inequality		
	Q1 least deprived (95% CI)	Q2 (95% CI)	Q3 (95% CI)	Q4 (95% CI)	Q5 highly deprived (95% CI)	SII	RII	*p*-value
All cancers combined C00-97 (excluding C44 and C77-79)								
Women	853 (848–858)	885 (880–891)	880 (875–886)	886 (881–891)	893 (887–899)	142	1.18	< 0.001
Men	1102 (1096–1108)	1212 (1205–1219)	1225 (1218–1231)	1250 (1244–1256)	1260 (1253–1268)	477	1.49	< 0.001
Mouth C00–06								
Women	9 (8–9)	9 (9–10)	9 (9–10)	10 (10–11)	10 (10–11)	6	1.84	0.001
Men	18 (17–18)	19 (18–20)	19 (18–20)	23 (22–24)	24 (23–25)	21	2.78	< 0.001
Stomach C16								
Women	20 (19–20)	21 (21–22)	21 (21–22)	22 (22–23)	23 (22–23)	7	1.39	0.011
Men	40 (39–42)	42 (41–43)	42 (41–43)	44 (43–46)	46 (44–47)	19	1.57	< 0.001
Colorectal C18-20								
Women	91 (90–93)	97 (95–99)	103 (101–104)	100 (98–101)	103 (101–105)	33	1.40	< 0.001
Men	136 (134–139)	150 (148–153)	158 (155–160)	159 (157–161)	162 (160–165)	69	1.57	< 0.001
Pancreas C25								
Women	34 (33–35)	36 (35–37)	34 (33–35)	36 (35–37)	35 (34–36)	9	1.27	0.026
Men	43 (42–44)	47 (45–48)	44 (43–46)	46 (45–48)	46 (44–47)	15	1.38	0.001
Lung C33-34								
Women	83 (81–85)	91 (89–93)	88 (86–89)	95 (93–96)	105 (103–107)	70	2.20	< 0.001
Men	135 (133–137)	156 (154–159)	167 (165–170)	178 (175–180)	197 (194–200)	165	2.75	< 0.001
Skin melanoma C43								
Women	41 (40–42)	45 (43–46)	44 (43–45)	37 (36–38)	37 (36–38)	−9	0.80	0.043
Men	58 (57–60)	59 (58–61)	55 (54–57)	50 (49–51)	47 (46–49)	−25	0.64	< 0.001
Breast C50								
Women	281 (278–283)	278 (275–281)	275 (272–278)	266 (263–269)	263 (260–266)	−17	0.94	0.061
Cervix Uteri C53								
Women	15 (14–16)	14 (14–15)	14 (13–15)	16 (15–16)	16 (15–17)	2	1.16	0.182
Prostate C61								
Men	296 (293–300)	327 (323–330)	326 (323–330)	312 (309–315)	302 (298–306)	14	1.04	0.420
Other cancers								
Women	283 (281–286)	291 (288–294)	303 (300–306)	295 (292–298)	303 (300–306)	64	1.25	< 0.001
Men	375 (372–379)	407 (403–411)	423 (420–427)	423 (419–427)	435 (430–439)	192	1.60	< 0.001

SII (Slope Index of Inequality) refers to the absolute distance between the most and least deprived district in the incidence accounting for the total variance in incidence across the socioeconomic deprivation spectrum of all districts; RII (Relative Index of Inequality) refers to the relative distance between the most and least deprived district in the incidence accounting for the total variance in incidence across the socioeconomic deprivation spectrum of all districts; the p-value corresponds to the respective cancer-specific regression model from which the values for the slope and the relative index of inequality were estimated

Data: annual epidemiological data from the Centre for Cancer Registry Data at the RKI, population statistics of the Federal Statistical Offices, GISD, own calculations

**Fig. 2:**
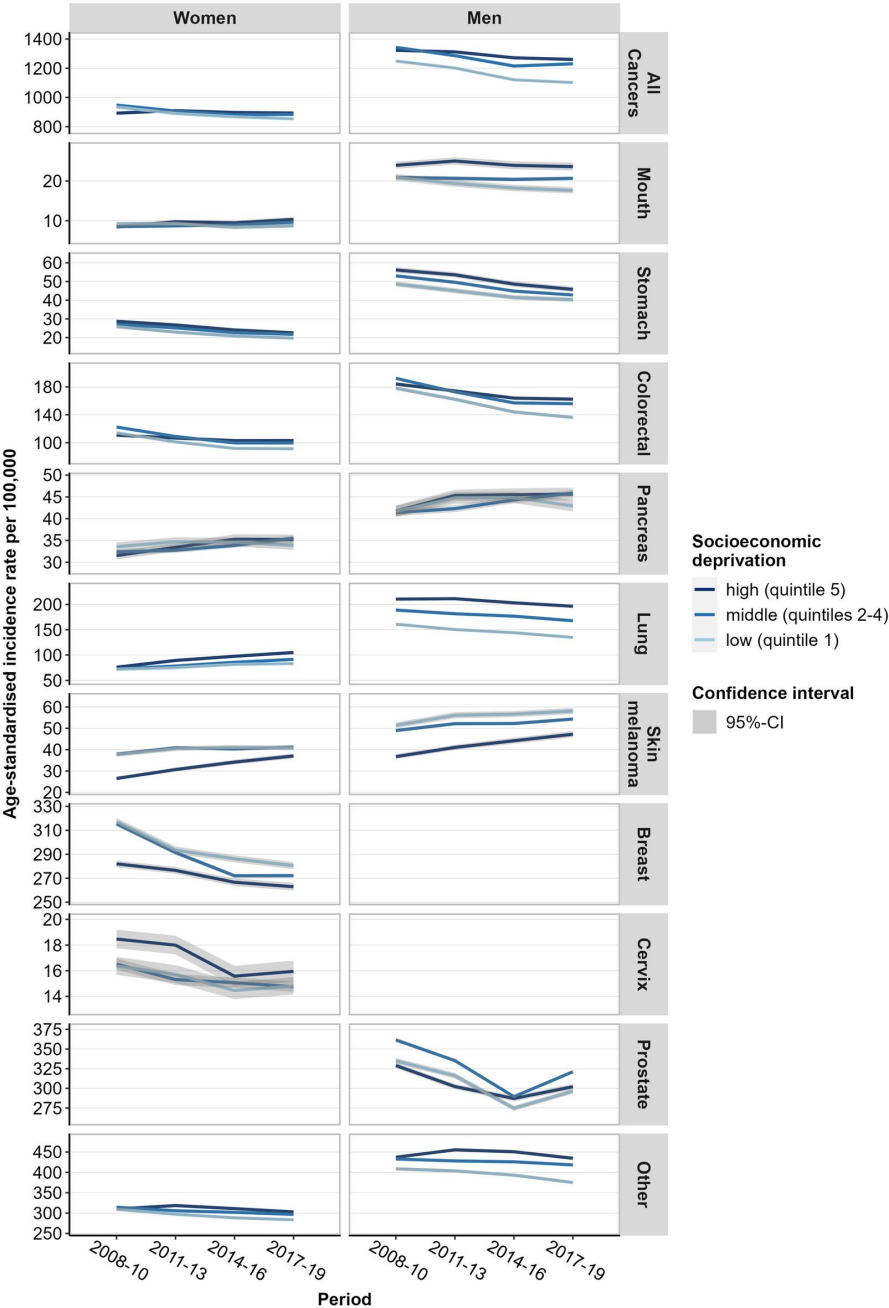
Time trends in annual age-standardised cancer incidence rates per 100,000 inhabitants at age 40 + by sex, area-level socioeconomic deprivation, and cancer entity, 2008–2019. Data: annual epidemiological data from the Centre for Cancer Registry Data at the RKI, population statistics of the Federal Statistical Offices, GISD, own calculations.

In women, the age-standardised incidence for all cancers combined decreased in mid-deprived and low-deprived areas, but increased in highly deprived areas over time (Fig. 2, Table S3-S5). Whereas substantial reductions in the age-standardised incidence were found for stomach, colorectal and cervical cancer among women in both more and less deprived areas, other trends were observed for cancers of the lung, mouth, pancreas, and in skin melanoma, the incidence of which increased especially in more deprived areas (Fig. 2, Table S3-S5). Among men, we observed a clear socioeconomic gradient for all cancers combined as well as for colorectal, stomach and lung cancer. For these site-specific cancers the incidence rates declined over time. Compared to women, the gradient in area-level socioeconomic deprivation was much more pronounced in men. Furthermore, we found among men decreasing age-standardised incidence rates for cancers of the mouth in districts with low deprivation but stagnating trends in those with middle and high deprivation. In all three deprivation groups, we found a downward trend in prostate cancer incidence until 2016 and an increase until 2019, while no clear gradient was found. The incidence of pancreatic cancer increased in both sexes in all groups of socioeconomic deprivation until 2011–2013, then stagnated or decreased slightly in low-deprived women and men. However, no area-level socioeconomic inequalities between groups of districts were identified for pancreatic cancer in both sexes. While the incidence of malignant melanoma of the skin in women and men increased in all deprivation groups, a clear reverse gradient was found with higher age-standardised incidence rates in districts with low socioeconomic deprivation (Fig. 2, Table S3-S5).

**Fig. 3:**
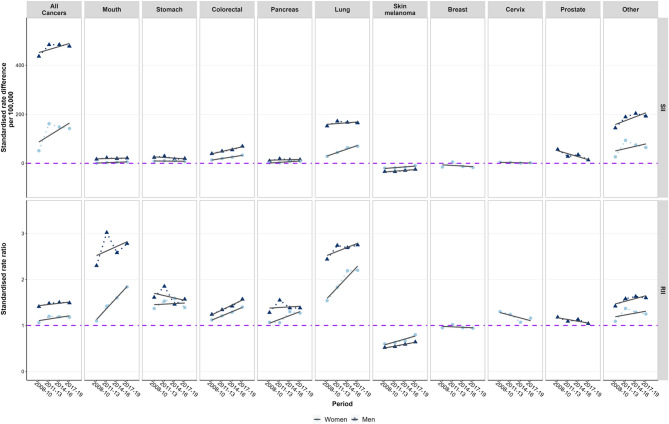
Absolute (SII) and relative (RII) inequalities in annual age-standardised cancer incidence between the most and least deprived district by sex and cancer entity, 2008–2019. Data: annual epidemiological data from the Centre for Cancer Registry Data at the RKI, population statistics of the Federal Statistical Offices, GISD, own calculations.

The regression-based analysis of trends in inequalities (SII and RII) by area-level socioeconomic deprivation revealed increasing inequalities in almost all cancer entities to the disadvantage of residents living in the most deprived districts (Fig. 3, Table S3-S5). This widening of inequalities was most pronounced in mouth, colorectal, pancreas and lung cancers in both sexes. For stomach cancer, the trend for men and women is ambiguous, but socioeconomic inequalities remain marked, with higher rates of new cases in more deprived districts. With regard to cervical and prostate cancer, inequalities between the most and the least deprived districts have been declining over time. In malignant melanoma of the skin inequalities have also decreased but remained visible through the end of the observation period (Fig. 3, Table S3-S5).

**Table 3:** Counterfactual scenarios of potential decreases in the annual number of cancer cases if area-level socioeconomic inequalities in cancer incidence could be reduced (what-if analysis) by sex and cancer entity in the period 2017–2019.

	Women				Men			
	Observed	Scenario 1	Scenario 2	Scenario 3	Observed	Scenario 1	Scenario 2	Scenario 3
**All cancers combined C00-97 (excluding C44 and C77-79)**								
Incident cases	566,871	549,382	561,815	564,900	648,804	590,271	631,160	641,622
Potentially reduceable incident cases		−17,489	−5,056	−1,971		−58,533	−17,644	−7,182
Proportion of potentially reduceable incident cases in observed incident cases		3%	1%	0%		9%	3%	1%
**Mouth C00–06**								
Incident cases	6,173	5,606	5,949	5,938	11,379	9,790	10,624	10,401
Potentially reduceable incident cases		−567	−224	−235		−1,589	−755	−978
Proportion of potentially reduceable incident cases in observed incident cases		9%	4%	4%		14%	7%	9%
**Stomach C16**								
Incident cases	14,493	13,308	14,105	14,248	22,893	21,454	22,235	22,228
Potentially reduceable incident cases		−1,185	−388	−245		−1,439	−658	−665
Proportion of potentially reduceable incident cases in observed incident cases		8%	3%	2%		6%	3%	3%
**Colorectal C18-20**								
Incident cases	66,630	61,623	65,301	66,123	81,875	72,945	79,118	81,185
Potentially reduceable incident cases		−5,007	−1,329	−507		−8,930	−2,757	−690
Proportion of potentially reduceable incident cases in observed incident cases		8%	2%	0%		11%	3%	1%
**Pancreas C25**								
Incident cases	23,865	22,995	23,638	23,563	24,224	22,858	23,829	23,849
Potentially reduceable incident cases		−870	−227	−302		−1366	−395	−375
Proportion of potentially reduceable incident cases in observed incident cases		4%	1%	1%		6%	2%	2%
**Lung C33-34**								
Incident cases	57,912	52,447	55,231	54,987	89,464	72,513	82,934	85,389
Potentially reduceable incident cases		−5,465	−2,681	−2,925		−16,951	−6,530	−4,075
Proportion of potentially reduceable incident cases in observed incident cases		9%	5%	5%		19%	7%	5%

Cancers with not clear social gradients (breast cancer, cervix uteri, prostate cancer and skin melanoma) were excluded from the what-if analysis, as their inclusion is not in accordance with the methodological assumptions of the what-if analysis (see statistical analysis section).

Data: annual epidemiological data from the Centre for Cancer Registry Data at the RKI, population statistics of the Federal Statistical Offices, GISD, own calculations.

According to our scenario-based what-if analysis (Table 3), 2017–2019 approximately 1–9% of all new cancer cases in men (annually PRIC = 7,182–58,533) and 0–3% in women (annually PRIC = 1,971 − 17,489) could potentially have been reduced if inequalities in incidence by area-level socioeconomic deprivation had been reduced (scenario 2 and 3) or eliminated (scenario 1). The analysis revealed that cancers of the lung, mouth, and colorectum have the greatest potential for reducing incidence by narrowing or eliminating area-level socioeconomic inequalities in the incidence. This holds true for both sexes. For instance, 5–19% (annually PRIC = 4,075–16,951) of all new lung cancer cases in men would have been reduced in 2017–2019. In women, around 9% (annually PRIC = 5,465) of lung cancer cases would be reduced in scenario 1. For colorectal cancer we found in the maximum scenario, 11% potentially reduceable incident cases among men (annually PRIC = 8,930) and 8% among women (annually PRIC = 5,007)). Comparable reduction potential to lung cancer in relative terms was also found for mouth cancer. If area-level socioeconomic inequalities could be eliminated or reduced, annual mouth cancer incidence rates would be reduced by 9–14% for men and 4–9% for women.

### Results sensitivity analysis

Despite the different assumptions between the main and sensitivity analyses, the case number distributions only varied slightly (Table S1). Therefore, the results were quite similar to those of the main analysis. This also held true for the patterns of inequality and trends in the cancers considered. Neither the omission of the most heterogeneous districts (Figure S1-S2) nor considering the level of socioeconomic deprivation 10 years before incidence (Figure S3-S4) influenced the results substantially.

## Discussion

The aim of our study was to depict the development of area-level socioeconomic inequalities in total and site-specific cancer incidence across Germany, and to determine how much lower the number of incident cancer cases could be if these inequalities were reduced or eliminated. Overall, the age-standardised incidence for all cancers combined, as well as for most site-specific cancers, declined over time. However, in most of the entities considered the declining trend was accompanied by widening inequalities, as incidence declined more strongly in less-deprived districts. This holds true for all cancers combined, stomach and colorectal cancer in both sexes as well as for lung cancer and cancers of the mouth in men. Furthermore, we found rising incidence rates and widening socioeconomic inequalities for lung cancer incidence in women. Taken together, these findings indicate that residents living in the socioeconomically more deprived districts of Germany did not benefit from positive trends and achievements in cancer prevention to the same extent as residents in socioeconomically less deprived areas. Exceptions, however, were malignant melanoma of the skin in both sexes and female breast cancer, where reverse socioeconomic gradients were observed with lowest incidence rates among residents of highly deprived districts. The study also reveals that the annual number of new cancer cases would have been approximately 9,100 to 76,000 cases lower in the period 2017–2019 if residents in more deprived areas had not been disadvantaged (or at least less disadvantaged) in terms of their cancer risk as compared to those in more affluent areas.

Reducing inequalities in favour of the socioeconomically more deprived population and the associated reduction in cancer could have positive and far-reaching social consequences. For example, the social differences in life expectancy between regions could be reduced, as area-level socioeconomic inequality in life expectancy is greatly driven by cancer^2^. Furthermore, cancer represents a considerable burden on the healthcare system^33^, which could be reduced by lowering the incidence. Strategies of cancer prevention not only intervening at the individual level, but also structurally at the community, local and societal levels by promoting healthy circumstances for all segments of the population (e.g. through smoke-free legislation, sugar taxation, occupational and environmental safety) should remain a central goal. With this in mind, reducing socioeconomic inequalities in cancer incidence should be defined as a key pillar in cancer prevention strategies and an important political goal. To achieve this, the Health in all Policies approach needs to be strengthened on the policy agenda. The findings of this study suggest that pursuing this goal may be very beneficial from a public health perspective and can make a significant contribution to reducing the incidence of cancer in Germany.

In line with our study, previous research based on health insurance data^12,17^ reported decreasing incidence rates for most cancers in Germany, which led to substantial gains in cancer-free life expectancy over time. Furthermore, growing socioeconomic inequalities were reported, which led to a growing difference in cancer-free life expectancy between individuals with low and high SEP. Supporting our findings, these studies show that widening inequalities in cancer incidence are also found based on individual-level socioeconomic information^12,17^, although these data are limited to the health insurance population.

A study based on cancer registry data from Germany’s western federal states also found widening inequalities in cancer incidence in those states^14^. The inequalities in incidence reported by Jansen et al. were, however, stronger than in our study. There are two possible explanations for this. Firstly, Jansen et al. used a different deprivation index which, in addition to the SEP dimensions, also contains social capital indicators^14^. As a result, their findings are not directly comparable to our study. The index used by Jansen et al. is not publicly available, so we were unable to carry out a comparative analysis. A second explanation could be that the regional differences in socioeconomic deprivation are masked by the regional distribution of cancer incidence between the districts in eastern and western Germany. For instance, the official statistics report a lower level of lung cancer in women in the eastern federal states where the highest levels of socioeconomic deprivation are observed^19^. In contrast, higher levels of lung cancer incidence in the western federal states were observed where districts with the highest level of socioeconomic deprivation were located. This pattern of the regional distribution of cancer incidence attenuates the socioeconomic gradient in lung cancer incidence in women.

For the most common cancers (e.g. lung cancer and colorectal cancer) the socioeconomic inequalities in their incidence among men and women found in our study were quite similar to those described earlier^8,12–14,17^. This is also true for the incidence of female breast cancer, for which we found a reverse association with area-level socioeconomic deprivation, which has also been reported for many high-income countries based on area-level socioeconomic deprivation or individual-level educational attainment^8^. Previous studies in Germany have also provided initial hints of this reverse association^12,13^. Unobserved risk factors (e.g. age at first pregnancy, number of children, no breastfeeding) for an increased risk of breast cancer incidence may contribute to explaining this association. Moreover, an association between low individual-level SEP, high regional socioeconomic deprivation, and higher incidence rates was also shown for the other cancers of the digestive system (stomach cancer and pancreatic cancer)^8,12,13^.

Our study also shows clearly widening socioeconomic inequalities in the incidence of all cancers combined over time with higher incidence rates among residents of areas with higher socioeconomic deprivation. Our study supplements previous research by showing the development over time and illustrating that area-level socioeconomic inequalities have been widening for most cancers in the past decades. A reverse gradient was found for melanoma of the skin, which is also in line with previous international literature^8,12,13^. A recent study reports clear but stable area-level socioeconomic inequalities in cervical cancer incidence between 2009 and 2019^23^. Another study for Germany, based on health insurance data, found no socioeconomic inequalities in the incidence risk of cervical cancer using individual income as a determinant of SEP^12^. In contrast, we found decreasing area-level socioeconomic inequalities in cervical cancer incidence over time. However, the previous studies for Germany used other study designs, a different cut-off age and standard population. Therefore, our results are not directly comparable to those previous studies.

Earlier studies describe the potential of cancer screening to reduce both mortality^34^ and health inequalities in cancer incidence in the long term, but cancer screening has been found to reach individuals with low SEP less frequently than people with higher SEP^35–39^. Assuming effective benefits of cancer screening, reducing socioeconomic inequalities in the utilisation of cancer screening has a certain potential to reduce socioeconomic inequalities in mortality, since cancer mortality is the largest contributor to socioeconomic inequalities in all-cause mortality, particularly among individuals aged 40–74 years^2^. Compared to primary prevention, the potential of secondary cancer prevention via screening to reduce health inequalities may be rather small. For example, there is no screening program for lung cancer, but lung cancer is a common cancer with a major contribution to earlier mortality among people in deprived circumstances^2^. Primary prevention with upstream approaches, e.g. in the field of tobacco control at societal level, seems therefore more likely to make significant contributions to reducing healthy inequalities. A recent study for Germany, based on official cause-of-death statistics, shows considerable area-level socioeconomic inequalities in mortality from all cancers considered in our study, with the exception of malignant skin cancer, where no inequalities in mortality were found^24^. These area-level socioeconomic inequalities in cancer mortality widened clearly between 2003 and 2019. One exception was stomach cancer in both sexes, where a convergence between districts of low and high socioeconomic deprivation in mortality was observed^24^. In general, similar patterns of socioeconomic inequality in incidence and mortality can be observed, particularly for cancers that are strongly associated with smoking. For instance, the inequality between most- and least-deprived districts in lung cancer mortality roughly doubled for both sexes between 2003 and 2019^24^. Although cancer mortality has decreased overall in recent decades, this decrease was not observed in the most deprived districts, or was much weaker than in the less deprived districts^1,2^. In this context, early cancer deaths, which occur before the age of 75, contributed most to the widening gap in socioeconomic inequalities in life expectancy^2^.

Behavioural (e.g. smoking^19,40–42^), obesity^19,43^, environmental (e.g. working environment^44^) and structural context factors (tobacco and alcohol control policy^45–47^) belong to the main drivers of socioeconomic inequalities in cancer incidence. Among them, smoking has been identified as explaining up to 61% of socioeconomic differences in a recent analysis in England^48^. It is well known that the importance of smoking for explaining socioeconomic inequalities decreases in men and increases in women, as the latter entered the tobacco epidemic at a later point in time^49,50^. The progression of the smoking epidemic with a later but then stronger adaptation of the harmful behaviour by individuals with low SEP could also be shown for Germany^51^. Recent observations of socioeconomic inequalities in smoking prevention among adults also suggest that the lung cancer incidence and mortality trends postulated in the smoking epidemic could continue in the future^42^. The shift in age-specific lung cancer incidence over time towards socioeconomically disadvantaged groups as a consequence of the smoking epidemic and its influence on lung cancer-free life expectancy has also been demonstrated for Germany^12,17,52^. Our study also found both a convergence between the sexes and a widening of socioeconomic inequality in the incidence of cancers of the lung and the mouth.

## Strengths and limitations

The study is based on the data from the official cancer registers. With an average of 450,000 cases annually, this represents the largest and most reliable data source on cancer incidence in Germany. Cases for which only a death certificate was available are also included in the data. In Germany, the registration of deaths is mandatory and independent of the reporting obligation of the treating institutions. Therefore, this approach has a positive effect on completeness and promotes a high validity of the cancer registry data used. The Centre for Cancer Registry Data at the RKI has processed the data according to international standards in order to increase data quality.

Another strength of the study is that our approach enabled us to analyse data from 2008 onwards for a large number of federal states (including eastern and western federal states) and to draw a more complete picture of the development of inequalities in cancer incidence in Germany than previous studies. However, during the last years of observation, the joint epidemiological cancer registry of the eastern German federal states was integrated into the existing clinical cancer registries of the respective federal states. Therefore, it cannot be ruled out that there may have been difficulties with the registration and late reporting of newly diagnosed cases during this transition phase. To address this limitation, we decided to include only those registries in our analyses that had an estimated completeness of approximately 90% across the three years of an observation period. If registers did not fulfil this criterion, they were excluded from the analyses, which applies to two registries in eastern Germany and one in western Germany (Saxony-Anhalt, Thuringia, Baden-Württemberg). The majority of the excluded districts are either at the bottom range of the GISD (in Saxony-Anhalt and Thuringia) or at the top (Baden-Württemberg). Due to this exclusion, we could not depict the full variation in area-level socioeconomic deprivation.

It can be assumed that the extent of cancer incidence in a region is at least partly a result of the socioeconomical conditions in the past. We therefore performed an additional sensitivity analysis to check the robustness of our results against this time-lag hypothesis. Here, using the GISD with a 10-year time lag, we found comparable levels, trends and inequalities in the site-specific incidence. This indicates that the results are not sensitive to the time lag applied. It is important to emphasise that, based on the data available for this study, we could not investigate causalities but associations and the extent of socioeconomic cancer inequalities across Germany. Thus, the counterfactual reduction of cancer incidence as suggested in the what-if scenarios might in fact be achieved by improving health-related lifestyles, which are fundamentally linked to material and non-material resources associated with economic deprivation^53^ in the sense of ‘causes of the causes’^54^. Our analyses are restricted to inequalities at the time of cancer incidence. As we have no information on the place of residence after incidence, we could not analyse the socioeconomic consequences of cancer. Furthermore, we used an ecological study design and therefore cannot rule out ecological fallacies. Yet, existing studies on socioeconomic inequalities in cancer incidence have consistently reported similar risk differentials, regardless of whether individual or area-based indicators are used^8^.

### Conclusions and implications

Our study shows that socioeconomic inequalities in the incidence of almost all cancers considered have been increasing in recent decades, and that people living in socioeconomically more deprived districts did not benefit to the same extent from the overall positive trend of decreasing cancer rates as people living in less deprived regions. Therefore, our study underlines the growing importance of cancer prevention for policies aiming to reduce health inequalities. Furthermore, the study provides information on how much lower the number of new cases could be for the most common cancers in Germany if residents in more deprived areas were not – or at least less – disadvantaged in terms of their cancer risks compared to those in more affluent areas. This underlines the importance of defining the reduction of socioeconomic inequalities in cancer incidence as an essential part of effective prevention policies^55^. Further studies are needed to identify relevant key factors that could explain the inequalities in specific cancer entities in more depth.

## Data Availability

The German cancer registry data analysed in this study are available on request from the German Centre for Cancer Registry Data at the Robert Koch Institute (http://www.Krebsdaten.de). The availability of these data is restricted due to legal reasons. The population numbers in five-year age groups for the German districts (Table 12411-04-01-4 and 12411-04-02-4) are freely accessible on GENESIS online (https://www.regionalstatistik.de/genesis/online/). The German Index of Socioeconomic deprivation is published under CC by 4.0 licence and is freely accessible via the GitHub website of the Robert Koch-Institute (https://github.com/robert-koch-institut) or on Zenodo^20^.
